# Metalloproteinases and their inhibitors—diagnostic and therapeutic opportunities in orthopedics

**DOI:** 10.3109/17453670903448257

**Published:** 2009-12-04

**Authors:** Björn Pasternak, Per Aspenberg

**Affiliations:** ^1^Orthopaedics and Sports Medicine, IKE, Linköping University, Linköping; ^2^Malmö University Hospital, Malmö, Sweden

## Abstract

Matrix metalloproteinases (MMPs) and related enzymes (ADAMs, ADAMTS) and their inhibitors control matrix turnover and function. Recent advances in our understanding of musculoskeletal conditions such as tendinopathy, arthritis, Dupuytren's disease, degenerative disc disease, and bone and soft tissue healing suggest that MMPs have prominant roles. Importantly, MMPs are amenable to inhibition by cheap, safe, and widely available drugs such as the tetracycline antibiotics and the bisphosphonates. This indicates that these MMP inhibitors, if proven effective for any novel indication, may be quickly brought into clinical practice.

## Introduction

Tissue-degrading enzymes of the metalloproteinase family have been implicated in the pathogenesis of several conditions involving the extracellular matrix. The family includes the matrix metalloproteinases (MMPs), their close relatives “a disintegrin and metalloproteinase” (ADAM), and “a disintegrin and metalloproteinase with thrombospondin motifs” (ADAMTS). MMPs are promising drug targets, and they are amenable to pharmacological inhibition by common clinically available drugs such as tetracyclines and bisphosphonates. A better understanding of the roles of metalloproteinases in tissue injury and the effects of their inhibition has the potential to improve outcome after injury and surgery. Additionally, metalloproteinases have diagnostic potential as markers to predict outcome of disease processes and healing, and to direct tailored therapy.

There has only been one published overview of the role of MMPs in orthopedics (Bramono et al. 2004), covering mostly arthritis. We extend the scope of that review by adding data from reports published in recent years and include a discussion on the ADAMs and ADAMTS, as well as conditions such as tendinopathy, Dupuytren's contracture, bone and soft tissue healing, and prosthetic loosening.

### Enzymes in control of the extracellular matrix

*Matrix metalloproteinases and their endogenous inhibitors.* Turnover of the extracellular matrix is a dynamic equilibrium between synthesis and degradation. Degradation is to a large extent mediated by matrix metalloproteinase (MMP) enzymes, which are antagonized by tissue inhibitors of metalloproteinases (TIMPs).

MMPs are a family of at least 24 zinc-dependent endopeptidases capable of degrading practically all components of the extracellular matrix (Table 1). MMPs contribute to many physiological processes through modification of the extracellular matrix (ECM) (Mandal et al. 2003). Recent insights suggest that MMPs may also have a broader spectrum of functions including regulation of the inflammatory response, for example through effects on chemokine and cytokine signaling and by release of neoepitopes from the ECM (Pearce and Shively 2006). MMPs have been subdivided according to their main (or first discovered) degradative activity, and the list of known substrates is continously growing. The collagenases (MMP-1, -8, and -13) are able to degrade practically all subtypes of collagen, most importantly the fibrillar collagens that provide mechanical strength to tissues. Collagenase 4 (MMP-18) is classically described as a *Xenopus* collagenase and is often omitted from lists of human MMPs. However, detectable levels of mRNA for this enzyme have been found in human ligaments (Foos et al. 2001). In addition to the classical collagenases, MMP-2 and MMP-14 (see below) also have important collagenolytic activity.

**Table 1.** The MMP family. Compiled from Hidalgo and Eckhardt (2001), Chakraborti et al. (2003), Pavlaki and Zucker (2003), Visse and Nagase (2003), Illman et al. (2006), Nagase et al. (2006), Pearce and Shively (2006). Stromelysin 3 is grouped with “other MMPs” since the enzyme has different properties from other stromelysins. Among the collagenous substrates for collagenases, bold numbers for collagens indicate strongest enzymatic activity

Group	MMP	Collagenous substrates	Noncollagenous ECM substrates	Nonstructural ECM component substrates
**Collagenases**				
Collagenase 1	MMP-1	collagens I, II, **III**, VII, VIII, X, XI, gelatins	proteoglycans, fibronectin, entactin, laminin, tenascin, vitronectin	α-1-antiprotease, pro-TNFα
Collagenase 2	MMP-8	collagens **I**, II, III, V, VII, VIII, X	fibronectin, laminin, proteoglycans	ADAMTS-1, pro-MMP-8
Collagenase 3	MMP-13	collagens I, **II**, III, IV, V, VII, IX, X, gelatins	proteoglycans, fibronectin, laminin, tenascin	fibrinogen, proMMP-9 and -13
**Gelatinases**				
Gelatinase A	MMP-2	gelatins, collagens I, II, III, IV, VII, X	laminin, elastin, fibronectin, proteoglycans	pro-MMPs -9 and -13, α-1-antiprotease, IGFBPs, IL-1β, TGFβ
Gelatinase B	MMP-9	gelatins, collagens IV, V, VII, X, XI	laminin, elastin, fibronectin, proteoglycans	α-1-antiprotease, CXCL5, IL-1β, TGFβ, plasminogen
**Stromelysins**				
Stromelysin 1	MMP-3	collagens III, IV, V, VII, IX, X, XI, gelatins	laminin, fibronectin, elastin, proteoglycans	pro-MMPs, pro-TNFα, E-cadherin, L-selectin, fibrinogen
Stromelysin 2	MMP-10	collagens I, III, IV, V, IX, X, gelatins	laminins, proteoglycans	pro-MMPs
**Matrilysins**				
Matrilysin 1	MMP-7	gelatins, collagens I and IV	laminin, elastin, fibronectin, proteoglycans, tenascin	pro-MMPs, pro-α-defensin, pro-TNFα, E-cadherin
Matrilysin 2	MMP-26	as above	as above	as above
**Membrane-type (MT) MMPs**				
MT1-MMP	MMP-14	gelatin, collagens I, II, III	proteoglycans, fibronectin, tenascin, fibrinogen	Pro-MMP-2 and -13
MT2-MMP	MMP-15	gelatins, collagen III		Pro-MMP-2
MT3-MMP	MMP-16		fibronectin	Pro-MMP-2
MT4-MMP	MMP-17			
MT5-MMP	MMP-24	gelatin	fibronectin	Pro-MMP-2
MT6-MMP	MMP-25			
**Other MMPs**				
Stromelysin 3	MMP-11		fibronectin	α-1-antiprotease, serpins
Metalloelastase	MMP-12	collagens, gelatins	elastin, proteoglycans	plasminogen
RASI	MMP-19		components of basement membranes	
Enamelysin	MMP-20		amelogenin	
-	MMP-21	gelatin		
-	MMP-23			
-	MMP-27			
Epilysin	MMP-28			Pro-TGFβ

ADAMTS: a disintegrin and metalloproteinase with thrombospondin motifs; ECM: extracellular matrix; TGF: transforming growth factor; TNF: tumor necrosis factor; RASI: rheumatoid arthritis synovial inflammation; IGFBP: insulin growth factor binding protein; CXCL: CXC chemokine ligand.

The gelatinases (MMP-2 and -9) degrade smaller collagen fragments, released during activity of the collagenases. Membrane-type MMPs (MT-MMPs) are cell membrane-linked proteases with diverse functions. Yet again demonstrating the complexity of the MMP system, MT1-MMP (MMP-14) is also released into the circulation (Ren et al. 2008). Stromelysins (MMP-3 and -10) and matrilysins (MMP-7 and -26) are broad-spectrum proteinases that also have important regulatory functions such as activation of other MMPs.

While most MMPs are secreted into the extracellular space immediately after synthesis as proenzymes (pro-MMP), some may also be stored within cells (e.g. MMP-9 in neutrophil granules), and others are bound to cell surface membranes (e.g. MT1-MMP). The pro-MMPs are activated by proteolytic cleavage in the extracellular space, where MMP-3 appears to be a key player by activating other MMPs in this manner (van Meurs et al. 1999, Dollery and Libby 2006). Baseline production of MMPs is low. Synthesis of MMPs is induced by a broad range of stimuli including cytokines (interleukin-1, -4, -6, and -10, and tumor necrosis factor-α), growth factors, EMMPRIN (extracellular MMP inducer) and cell-cell or cell-matrix interactions, which all signal through intracellular pathways such as the mitogen-activated protein kinase pathway (Kossakowska et al. 1999, Meller et al. 2000, Hidalgo and Eckhardt 2001, Corps et al. 2004, Gabison et al. 2005).

**Figure 1.**
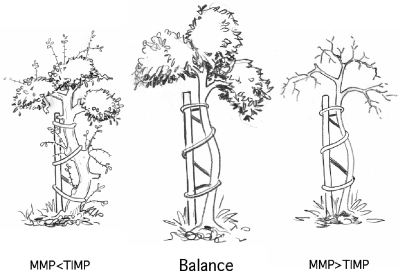
Degradation of the extracellular matrix is principally mediated by MMPs, which are counterbalanced by TIMPs. Disturbances of this equilibrium may lead to disease processes of fibrotic nature (left) or degradative nature (right).

**Figure 2.**
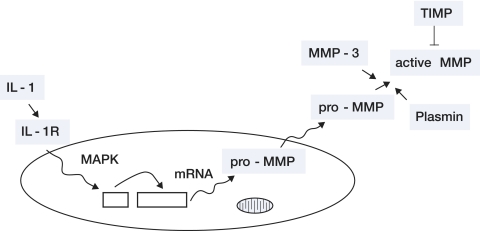
Simplified drawing of MMP regulation. MMP gene transcription is generally induced by stimuli such as inflammatory cytokines, which signal via specific intracellular pathways. MMPs are produced as inactive pro-enzymes that are subsequently cleaved to become active enzymes. MMP-3 appears particularly important in this regard, since it is known to activate several of the MMPs. Plasmin is also an important activator of MMPs. TIMPs inhibit MMPs mainly at the active level. MAPK: mitogen-activated protein kinase.

The composition of the ECM depends on the balance between tissue formation and breakdown (Figure 1). Because the latter is mediated mostly by MMPs, strict regulation of MMP production and activity is an essential part of ECM homeostasis. This regulation takes place at the levels of gene transcription, pro-MMP activation, and inhibition of active enzymes (Figure 2).

There are 4 TIMPs, which reversibly inhibit all MMPs by 1:1 interaction with the zinc-binding site (Hidalgo and Eckhardt 2001). TIMP-1, -2, and -4 are found in the tissues as well as in the circulation, while TIMP-3 is sequestered in the ECM (Baker et al. 2002). The specificity of TIMPs for individual MMPs is quite overlapping, although MT-MMPs appear to be resistant to TIMP-1 (Baker et al. 2002, Dollery and Libby 2006). TIMPs have several functions besides MMP inhibition, such as their roles in regulation of angiogenesis and cellular proliferation (Chirco et al. 2006). Additional endogenous inhibitors of MMPs include the soluble α-1-antitrypsin and α-2-macroglobulin (Nagase et al. 2006), as well as cell membrane-linked MMP inhibitors (Baker et al. 2002).

MMPs appear to have prominent roles in several diseases with a component of tissue destruction, such as osteoarthritis and rheumatoid arthritis (Bramono et al. 2004). It should be mentioned, however, that the view of MMPs as being solely tissue-degrading enzymes is somewhat oversimplified. This has been exemplified in a model of *Porphyromonas gingivalis*-induced periodontitis, where MMP-8 knockout mice suffered a more extensive alveolar bone loss than wild-type mice (Kuula et al. 2009). These findings may be explained by data showing that MMPs can exert anti-inflammatory actions, possibly by processing of anti-inflammatory cytokines and chemokines (Owen at al. 2004, Gueders et al. 2005). The variable roles of MMPs are further illustrated by data from a case study on patients with a phenotype of multicentric osteolysis with e.g. carpal and tarsal resorption, arthritis, and osteoporosis. These patients originated from consanguinous families, and were shown to have a complete absence of pro- and active MMP-2 in serum, explained by 2 specific mutations in the MMP-2 gene (Martignetti et al. 2001). This underscores the complex physiological and developmental roles of MMPs.

*ADAMs and ADAMTS.* Two other groups of proteases are related to the MMPs. ADAMs are cell membrane-linked enzymes with proteolytic and cell signaling functions. ADAMTSs are secreted into the circulation and constitute a heterogenous family of proteases with both anabolic and catabolic functions.

“A disintegrin and metalloproteinase” (ADAM) and “ADAM with thrombospondin motifs” (ADAMTS) proteins belong to the family of metalloproteinases. Only recently discovered, their functions are only beginning to be unravelled. ADAMs are usually linked to the cell membrane while ADAMTS are secreted pericellularly and into the circulation. However, alternate splicing of the ADAM genes also gives rise to circulating forms of these proteins. There are at least 33 ADAMs, and their functions include pericellular proteolysis of other membrane-bound proteins (e.g. growth factors in precursor state), cell signaling, and cell adhesion (Mochizuki and Okada 2007). The 19 known subtypes of ADAMTS are divided into 4 groups. The aggrecanases (ADAMTS-1, -4, -5, -8, -9, -15, and -20) can be roughly described as having proteoglycanolytic action, although there are other reported functions such as regulation of angiogenesis and degradation of other proteins for some of the group members. ADAMTS-2, -3, and -14 constitute the second group and they have anabolic function, acting as procollagen N-propeptidases for collagen types I, II, and III. Their importance in physiology is illustrated by the finding of an ADAMTS-2 mutation in Ehlers-Danlos syndrome type VII C (a condition with fragile skin, joint laxity, and hernias). Thirdly, ADAMTS-13 cleaves the von Willebrand factor involved in coagulation, and mutations in this protein are coupled to thrombotic thrombocytopenic purpura. There is a fourth group of ADAMTS (-6, -7, -10, -12, -16, -17, -18, and -19) with largely unknown function (Jones and Riley 2005). TIMP-3 is the main TIMP to have any inhibitory activity against some of the ADAMs and ADAMTS (Mochizuki and Okada 2007).

*MMP inhibitors.* There are several types of MMP inhibitors, including tetracycline antibiotics. If proven effective for any anti-MMP indication, these already approved drugs might be quickly be brought into clinical practice.

**Figure 3.**
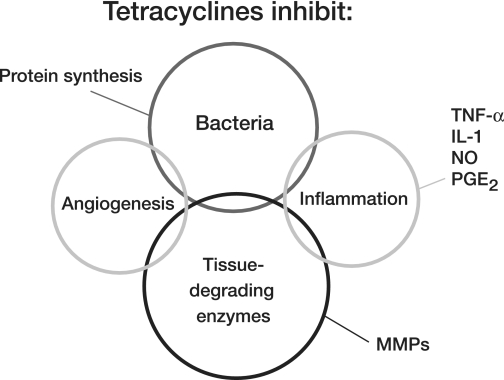
Mechanisms of action of tetracyclines. Tetracyclines have several non-antimicrobial effects. The ability of these drugs to inhibit matrix metalloproteinases is well established. Based on this mechanism, tetracyclines have shown clinical effects on rheumatoid arthritis and periodontitis. Doxycycline and minocycline are the two tetracyclines that have been studied most extensively. TNF: tumor necrosis factor; IL: interleukin; NO: nitric oxide; PG: prostaglandin.

There are several classes of pharmacological MMP inhibitors. The most common mechanism of action is binding to the zinc site of the MMP enzyme, thereby blocking its activity. The tetracycline antibiotics have several important non-antibiotic mechanisms (Figure 3) (Tamargo et al. 1991, Amin et al. 1996, Kloppenburg et al. 1996, Golub et al. 1998, Yrjanheikki et al. 1999, Lee et al. 2004, Sapadin and Fleischmajer 2006). Doxycycline is considered to be the most potent MMP inhibitor among the tetracyclines, and shows a broad spectrum by inhibiting MMPs -1, -2, -7, -8, -9, -12, and -13, although activity against the entire MMP family has not been determined (Golub et al. 1998, Peterson 2004). Besides zinc-binding, tetracyclines also inhibit MMPs at the gene expression level and by reducing activation via the inflammatory cascade and through reactive oxygen species.

Chemically modified tetracyclines have been developed to avoid unwanted effects on the endogenous microbial flora while retaining the MMP-inhibitory action. Effects on the microbial flora can also be avoided by administering “low-dose” doxycycline, i.e. 20 mg twice a day instead of the standard dose of 100 mg twice a day. In this way, doxycycline still retains its MMP-inhibitory efficacy (Emingil et al. 2004, O'Dell et al. 2006) but has no effect on the microbial flora of the vagina or the gut (Walker et al. 2005).

Synthetic MMP inhibitors constitute a large heterogeneous group of compounds, which have been modified to increase inhibitory potency and to produce increased specificity against particular MMPs (Hu et al. 2007). Bisphosphonates, known inhibitors of osteoclastic bone resorption, also have potent MMP-inhibitory properties, probably through cation-chelation of zinc (Hidalgo and Eckhardt 2001, Heikkila et al. 2002).

### Proteinases in soft tissues

*Tendon disease.* Tendon degeneration and rupture is largely mediated by MMPs. Inhibition may become a therapeutic alternative.

The typical histopathological finding in painful tendinopathy is degeneration, i.e. disorganized ECM, variation in the density of tendon cells ranging from hyper- to hypocellularity, and increase in vascularity (Riley 2004, 2008, Rees et al. 2006). Several reports have indicated that excessive matrix degradation has a role in the etiopathogenesis of painful tendinopathy and tendon rupture (Table 1) (Riley 2004, de Mos et al. 2007). One pilot study has also suggested that there is a systemic disturbance in the regulation of MMPs and TIMPs in tendon rupture (Pasternak et al. 2008b). The impact of systemic factors is emphasized by a study showing an association between variants of the MMP-3 gene and painful Achilles tendinopathy (Raleigh et al. 2009). Painful tendinopathy and tendon rupture are probably two somewhat distinct entities, since degeneration in ruptured tendons is generally painless but more severe than in painful tendinopathy (Tallon et al. 2001) and there are differences in the expression of structural proteins and proteolytic enzymes (Corps et al. 2006, Jones et al. 2006), as well as genetic differences (Mokone et al. 2006, Raleigh et al. 2009).

Enzyme inhibitors have not been subject to any extensive evaluation in the treatment of tendinopathy. 2 small trials with peritendinous injections of aprotinin, a general protease inhibitor, have been conducted and showed some benefit for patellar and Achilles tendinopathy (Magra and Maffulli 2005). Further support for its efficacy comes from a large case series on chronic tendinopathy (Orchard et al. 2008). However, in a small randomized trial on Achilles tendinopathy, treatment with aprotinin as an adjunct to eccentric training was not found to be better than placebo (Brown et al. 2006).

Pain in tendinopathy might be mediated by the neurotransmitter substance P (SP) (Alfredson 2005, Schubert et al. 2005). Interestingly, SP is capable of regulating the gene expression of MMPs and TIMPs in fibroblasts (Cury et al. 2008), which may link SP to the altered regulation profile of MMPs and TIMPs observed in tendinopathy. SP is also involved in repair processes and, when administered exogenously, appears to enhance proliferation of fibroblasts and tendon healing (Ackermann et al. 2003, Burssens et al. 2005, Steyaert et al. 2006). Furthermore, patients who develop shoulder stiffness after arthroscopic rotator cuff repair have been found to have markedly elevated plasma SP levels as compared to patients with a good postoperative outcome (Franceschi et al. 2008).

The Achilles tendon has served as the model tendinopathy for etiopathologic studies, and will probably serve as a model tendinopathy for treatment studies. However, the molecular profiles of tendinopathy in other regions should be determined since supraspinatus tendinopathy, for example, may be a distinct entity as compared to Achilles tendon disease (Lo et al. 2004). Thus, detailed molecular studies are required in order to be able to tailor specific mechanism-based therapy.

**Table 2.** mRNA expression and enzyme activity (or levels of active enzyme) of MMPs, TIMPs, ADAM, and ADAMTS in human tendon disease. Only proteins that showed statistically significant differences between groups are shown. Note that there are some contrasting results due to differences between studies. Data from Riley et al. (2002), Alfredson et al. (2003), Lo et al. (2004), Voloshin et al. (2005), Jones et al. (2006), de Mos et al. (2007), Corps et al. (2008), Karousou et al. (2008), and de Mos et al. (2009)

		Elevated	Decreased
Painful Achilles tendinopathy compared to control	Gene expression	MMP-2, -11, -13, -16, -23 ADAM-12 ADAMTS-2, -3	MMP-3, -10, -12, -27 TIMP-3 ADAMTS-5
	Activity	MMP-3	n.a.
Ruptured Achilles tendon compared to control	Gene expression	MMP-1, -2, -9, -11, -14, -17, -19, -25 TIMP-1 ADAM-8, -12 ADAMTS-4	MMP-3, -7, -24, -28 TIMP-2, -3, -4 ADAMTS-7, -13
	Activity	MMP-2 and 9 ADAMTS-4	n.a.
Ruptured rotator cuff compared to control	Gene expression	MMP-13	MMP-3 TIMP-2, -3, -4
	Activity	MMP-1, -9, -13	MMP-2

n.a.: not applicable.

*Tendon healing.* General inhibition of MMPs impairs tendon healing, but more selective treatment might possibly have a positive effect. Sutures coated with MMP inhibitors have a better grip.

Only a few studies have dealt with the role of MMPs in tendon healing. Based on mRNA data from unloaded healing rat flexor tendons, it has been suggested that MMP-9 and MMP-13 participate only in tissue degradation during the early phase of healing, while MMP-2, -3, and -14 participate both in tissue degradation and in later remodeling (Oshiro et al. 2003). These data must be confirmed—especially in view of the large differences observed in mechanical properties and expression of growth factor genes, depending on the loading status of the tendon during healing (Virchenko and Aspenberg 2006, Eliasson et al. 2008). An experimental study showed that systemic treatment with the MMP inhibitor doxycycline led to weakening of rat Achilles tendons during healing, which suggests that MMPs are important for tendon healing (Pasternak et al. 2006). However, the results do not rule out the possibility that MMP inhibitors could be used to enhance tendon healing, since studies of specific MMPs that act during tendon healing may identify those that are deleterious to healing, thus allowing treatment with inhibitors specific for certain subspecies of MMPs. Results from a pilot study suggest that the target MMP may be MMP-7, since there was a strong inverse correlation between levels of this proteinase and tendon strength during healing in humans (Pasternak et al. 2008b). MMP-13 could be another target; it has been implicated in tendon degradation caused by stress-deprivation (Arnoczky et al. 2007) and its expression is strongly upregulated in rotator cuff rupture (Lo et al. 2004).

Several experimental studies have shown that the mechanical properties of tendon can be manipulated by MMP inhibitors. An ex vivo rat tail tendon model showed that weakening of tendon induced by unloading could be inhibited by the MMP inhibitors GM 6001 and doxycycline (Arnoczky et al. 2007). Furthermore, a rabbit model of anterior cruciate ligament reconstruction showed that the endogenous MMP inhibitor α-2-macroglobulin, injected intra-articularly, improved mechanical and histological characteristics of tendon to bone healing (Demirag et al. 2005).

Experimental studies have shown that the strength of the suture construct in tendons becomes transiently reduced in the immediate postoperative period after surgical repair of tendon rupture (Wada et al. 2001, Yildirim et al. 2006). A degradative process, mediated by MMPs, is thought to occur in the direct vicinity of the sutures, leading to weakening of tendon tissue around the suture (McDowell et al. 2002). This is thought to facilitate the suture to cut through the tendon when exposed to tensile stress, i.e. during mobilization. Inhibition of MMPs could thus help to improve tendon suture-holding capacity. Based on this idea, it has been shown that doxycycline-coated sutures can improve early tendon suture-holding capacity in a rat model (Pasternak et al. 2007).

*Frozen shoulder.* Reduced MMP activity may be involved in the pathogenesis of frozen shoulder.

In the late 1990s, several cancer trials with MMP inhibitors were conducted. In one study, frozen shoulder and/or Dupuytren's-like contracture was reported in 6 of 12 gastric cancer patients treated with the MMP inhibitor marimastat (Hutchinson et al. 1998). Musculoskeletal side effects were later confirmed by reports of adverse effects from large trials evaluating MMP inhibitors for various indications. The group of side effects included frozen shoulder, Dupuytren's-like contracture, and arthralgias, and it was named the musculoskeletal syndrome (MSS). Although the exact mechanism is unknown, MSS is attributed either to the inhibition of too wide a spectrum of MMPs, or to unavoidable side effects of inhibiting the specifically targeted MMPs (Peterson 2004). Side effects from MMP inhibitors appear to be dose-dependent and develop only in patients on long-term therapy. The latter indicates that MSS will not be a problem for potential short-term indications, e.g. perioperative treatment. Furthermore, MSS is not a feature of every MMP inhibitor and has not been reported with tetracyclines. In addition, subspecies-selective MMP inhibitors may not have these side effects.

A non-quantitative gene expression study on frozen shoulder tissue showed an absence of MMP-14 as well as reduced expression of MMP-1, indicating that reduced collagenolysis may have a role in the pathogenesis of this disease (Bunker et al. 2000). Further studies using quantitative PCR and protein detection techniques will be needed in order to obtain a better understanding of this disease. Future therapy could possibly be directed at inhibition of the TIMPs, preferably by a local drug delivery approach.

*Dupuytren's disease.* This disease has a complex regulation profile of metalloproteinases and inhibitors. TIMP-1 may be the most important. Local injection of MMPs has shown therapeutic promise.

Dupuytren's disease of the hand, which is common in northern Europe, is a fibroproliferative disease of the palmar fascia. Myofibroblasts are responsible for contraction of the tissue, leading to hand dysfunction. Nodules in Dupuytren's disease represent active disease with myofibroblast proliferation, while cords are typical of late-stage disease. A comprehensive gene expression profiling of MMPs, TIMPs, and ADAMTS using tissue samples from nodules and cords of patients with Dupuytren's disease and control palmar fascia samples from patients with carpal tunnel disease revealed a complex regulation profile with distinctive patterns for active and late-stage disease (Table 3) (Johnston et al. 2007). Based on these findings, the authors suggested that contraction and fibrosis in Dupuytren's disease may result from several mechanisms including ADAMTS-14-mediated promotion of collagen synthesis, TIMP-1 inhibition of collagenolytic activity, and MMP-14-mediated pericellular collegenolysis, allowing contraction. Interestingly, levels of some of the MMPs and ADAMTS were found to show a correlation with recurrence more than a year later (Johnston et al. 2008), suggesting their possible clinical use as predictive markers. In addition, it was shown that the broad-spectrum MMP inhibitor GM6001 (ilomastat) reduced Dupuytren's disease fibroblast-mediated contraction in an in vitro model (Townley et al. 2008).

**Table 3.** mRNA expression of MMPs, ADAMTS, and TIMPs in nodule tissue (active disease) and cord tissue (end-stage disease) from patients with Dupuytren's disease (n = 19) as compared to control palmar fascia from patients with carpal tunnel syndrome (n = 19). Data from Johnston et al. (2007). Only proteins that showed statistically significant differences between groups are shown

	Elevated	Decreased
Dupuytren's nodule compared to control	MMP-1, -2, -7, -11, -13, -14, -15, -16, -17, -19, -21 ADAMTS-2, -3, -4, -5, -9, -14, -16, -18 TIMP-1	MMP-3 ADAMTS-1, -6, -8, -19, -20 TIMP-2, -3, -4
Dupuytren's cord compared to control	MMP-2, -7, -11, -13, -16 ADAMTS-3, -12, -18	MMP-3
Dupuytren's nodule compared to cord	MMP-2, -11, -13, -14, -15, -16, -17, -19, -28 ADAMTS-2, -4, -5, -12, -14 TIMP-1	MMP-3, -8 ADAMTS-1, -6, -8 TIMP-2, -4

One further study has highlighted the key role of TIMP-1 in the pathogenesis of fibrotic changes in Dupuytren's disease (Ulrich et al. 2003). The investigators showed elevation of TIMP-1 in the serum of patients with active Dupuytren's disease as compared to carpal tunnel syndrome controls, whereas the levels were similar between patients with inactive late-stage disease and controls. There was also a shift in the systemic MMP-TIMP balance. Detection of these changes in blood samples illustrates the important role of TIMP-1. In view of the strong hereditary component and a predilection for men in Dupuytren's disease, it is interesting to note that TIMP-1 is located on the X-chromosome. One approach to address this issue would be to investigate whether single nucleotide polymorphisms and other genetic variation in, for example TIMP-1, might explain some of the familial predisposition to Dupuytren's disease.

In view of TIMP-1 being an important mediator in the pathogenesis of Dupuytren's disease, pharmacological targeting of this protein has been suggested as a possible therapy (Johnston et al. 2007). In another approach to non-surgical therapy, the effect of collagenase injections has been investigated in a double-blind randomized placebo-controlled trial of 35 patients with Dupuytren's disease (Badalamente and Hurst 2007). Improvement was seen in 21 of 23 patients treated with up to 3 injections of collagenase, as compared to none of the patients treated with placebo. Although there are safety issues because of frequent adverse events, these results are promising and merit further investigation.

*Disorders of the spine.* Metalloproteinases are dysregulated in disc disease, and idiopathic scoliosis, ankylosing spondylitis, and disc disease have been connected to genetic variants of MMPs.

Degeneration of the anulus fibrosus of intervertebral discs, leading to disc herniation and sciatic pain, is thought to be mediated (at least in part) by MMP-1 and -3 (Goupille et al. 1998). Furthermore, a recent study has shown elevation of ADAMTS-1, -4, -5, and -15 in degenerated intervertebral discs, with the levels of ADAMTS-4, -5, and -15 increasing with increasing degree of degeneration (Pockert et al. 2009).

Degeneration of intervertebral discs appears to have a strong genetic component, as shown by epidemiological studies (Ala-Kokko 2002). This may be partly explained by MMP gene variants. A single nucleotide polymorphism (C to T) in the promotor region of the MMP-2 gene results in reduced transcription. Patients with lumbar disc degeneration have a higher rate of homozygosity for the CC genotype (Dong et al. 2007). Furthermore, patients with the the CC genotype have more severe disc degeneration than patients with CT and TT genotypes. This suggests that patients prone to disc degeneration have a tendency to have higher transcription of the MMP-2 gene and that among patients with disc degeneration, those with higher transcription of the MMP-2 gene have more severe disease. Whether or not the CC genotype is associated with higher tissue and systemic levels of MMP-2 is, however, unknown.

There is a highly variable region within the promoter region of the MMP-3 gene, the so-called 5A/6A polymorphism. One allele has a run of 6 adenosines (6A) and the variant allele has a run of 5 adenosines (5A). It has been reported that the 5A allele has twice as much promoter activity as the 6A allele. About half of the population carries this allele. In a radiographic study of volunteers, this genotype was associated with intervertebral disc degeneration in elderly, but not young, patients (Takahashi et al. 2001). In a small study, Aulisa et al. (2007) reported an association between adolescent idiopathic scoliosis and the 5A/6A MMP-3 promoter polymorphism. The rate of having a homozygous genotype for the 5A allele of the MMP-3 promoter was found to be 3 times higher in scoliotic patients (30%) than in healthy controls (11%).

MMP-3 has also been implicated in ankylosing spondylitis. Serum MMP-3 levels were found to be twice as high in ankylosing spondylitis patients than in controls, and showed a positive correlation with disease activity (Chen et al. 2006). In addition, serum MMP-3 was found to be an independent predictor of radiographic progression 2 years later, particularly in patients with pre-existing radiographic damage (Maksymowych et al. 2007). Circulating MMP-3 levels were also reduced by TNF-α-blocker treatment in ankylosing spondylitis, and there was a correlation between changes in MMP-3 and changes in disease activity during treatment (Maksymowych et al. 2008).

*Fracture healing.* Fracture non-union is associated with altered levels of serum MMPs. Variation in the healing of other tissues appears to be related to MMPs. This may also be the case for bone fractures, indicating that there may be therapeutic possibilities.

Patients who develop non-union of long bone fractures have been found to have higher levels of pro-MMP-1 and MMP-8, and lower TIMP-1 in serum during postoperative monitoring than patients who heal normally (Henle et al. 2005). The same research group also showed suppression of serum TGF-β in patients who developed non-union (Zimmermann et al. 2005). This suggests that there may be a possibility of monitoring and predicting the outcome of healing using blood samples. In fact, there have been a few studies in other fields of research that have shown associations between MMP levels and outcome of tissue healing. Preoperative MMP-9 levels in nasal secretions were found to show an inverse correlation with quality of healing after sinus surgery (Watelet et al. 2004), while MMP-9 in wound fluid obtained at 24 hours after inguinal hernia surgery showed an inverse correlation with collagen deposition at 10 days (Agren et al. 1998). MMP-1, -2, and -9 levels in peroperative intestinal biopsies were elevated in patients who developed anastomotic wound failure after colorectal resection (Stumpf et al. 2005). MMP inhibitors have been shown to improve healing of intestinal and cutaneous wounds (Witte et al. 1998, Syk et al. 2001, Pasternak et al. 2008a). Future experimental studies will show whether there is any effect of MMP inhibitors (general and selective) on fracture healing.

*Prosthetic loosening.* Genetic variants of MMP regulation are related to increased risk of loosening.

The causes of aseptic loosening of hip prostheses have been debated extensively (Sundfeldt et al. 2006). Studies of the implant interface membrane tissue have shown evidence of MMP-mediated collagen breakdown (Takagi 1996) and the extracellular matrix metalloproteinase inducer (EMMPRIN), which upregulates several MMPs, has been found at elevated levels around loosened implants (Li et al. 1999). Expression of several MMPs appears to be upregulated in macrophages in vitro in response to titanium and PMMA particles (Nakashima et al. 1998). These data suggest the possibility of pharmacological intervention in patients who (begin to) develop aseptic loosening. Intriguingly, in vitro bone resorption by cells obtained from the interface membrane of patients with aseptic loosening of hip prostheses can be inhibited by doxycycline (Ong and Taylor 2003).

Finally, a single nucleotide polymorphism (SNP) in the promoter region of the MMP-1 gene has been found to be associated with aseptic loosening of hip prostheses (Malik et al. 2007). Another, smaller study found an association between a different SNP in the MMP-1 gene promoter region and aseptic loosing of hip prostheses (Godoy-Santos et al. 2009). This SNP increases the activity of the MMP-1 gene (Rutter et al. 1998), and therefore patients with this gene variant may have elevated MMP-1 production that results in excessive tissue damage. Further investigation of this SNP may provide a way of identifying patients at risk of prosthetic loosening, even before surgery.

### Joint diseases

*Rheumatoid arthritis.* Because MMPs mediate joint destruction, MMP inhibitors are now used as therapeutics for mild rheumatoid arthritis.

MMPs have been established as major mediators of the destruction of joint cartilage and bone in rheumatoid arthritis (RA) (Tchetverikov et al. 2004, Rengel et al. 2007) and this knowledge is now about to be applied in the clinic. Circulating MMP-1 and -3 have been suggested to be promising markers of disease severity in RA (Green et al. 2003). One study in which several biomarkers were evaluated showed that baseline serum MMP-3 is a reliable predictor of radiographic progression of RA 2 years later (Young-Min et al. 2007). A functional polymorphism in the MMP-3 gene promoter, associated with elevated serum levels of MMP-3, has been linked to radiographic progression of RA in European but not in Japanese patients (Constantin et al. 2002, Mattey et al. 2004, Tsukahara et al. 2008).

Further evidence for the implication of MMPs in RA has come from treatment studies. 6 months of treatment with methotrexate of patients with newly diagnosed RA led to reduced serum levels of MMP-1, -9, -13, and TIMP-1 (Fiedorczyk et al. 2006). In another study, lefluonomide and methotrexate treatment both led to a decrease in activated MMPs in serum of patients with RA (Tchetverikov et al. 2008). Corticosteroid injections into the knee joint of patients with active RA synovitis induced suppression of serum MMP-3, but not of the MMP-1/TIMP-1 complex (Weitoft et al. 2008).

Tetracycline MMP inhibitors have been evaluated in a few randomized controlled RA trials. A study involving 66 patients with early seropositive RA evaluated doxycycline as an adjunct therapy to methotrexate. At the 2-year follow-up, the doxycycline-methotrexate combination had improved the main outcome variable (American College of Rheumatology; ACR 50%) as compared to methotrexate alone (O'Dell et al. 2006). Importantly, the positive effects were similar between the standard dose of 100 mg twice daily and 20 mg twice daily, corroborating the concept of low-dose doxycycline, i.e. that the mechanism of action is dependent on the MMP-inhibitory and anti-inflammatory actions of doxycycline rather than any possible antimicrobial effects. Benefit of another tetracycline MMP inhibitor, minocycline, has also been demonstrated in a randomized trial (O'Dell et al. 2001). There have, however, also been studies that showed no effects of tetracyclines on symptoms and progression of RA (van der Laan et al. 2001). Minocycline is currently included in the ACR guidelines as an alternative in the treatment of RA of short duration, with low disease activity and absence of poor prognostic features (Saag et al. 2008). There have been no published trials evaluating synthetic MMP inhibitors in RA. Because of the important pathophysiological roles of, for example, MMP-3 and -13 in RA, trials with selective MMP inhibitors are eagerly awaited.

When approaching the clinical use of MMP as markers of, for example, disease activity, there will be a need for standardization of sampling procedures and types of assay, as variations in levels between serum and plasma and several other factors are important for the interpretation of the results (Mannello 2008).

*Osteoarthritis.* MMP-3 levels appear to be related to prognosis. MMP inhibitor therapy has already been tried, with mixed results.

Gene expression for several of the proteolytic MMP and ADAMTS enzymes is upregulated in cartilage and synovium in patients with osteoarthritis (OA) (Davidson et al. 2006). Furthermore, there is downregulation of TIMP-1 and -4 expression in OA cartilage, suggesting an altered balance favoring proteolysis. MMP-13, the collagenase with the strongest activity against type II collagen, is thought to be one of the key metalloproteinases in OA-associated joint destruction (Takaishi et al. 2008).

MMP-3 has been evaluated as a prognostic tool in prediction of the disease course in OA. Patients with plasma MMP-3 levels in the upper tertile of the baseline distribution have been found to be more likely to suffer progression of OA over a 30-month period than patients in the lower tertile (odds ratio: 4) (Lohmander et al. 2005).

Randomized trials have shown conflicting results of MMP inhibitor treatment in OA. Doxycycline has been found to have positive effects on radiographic progression but had no effect on joint pain (Brandt et al. 2005), while PG-116800, a broad-spectrum synthetic MMP inhibitor, showed no effect in a large study (Krzeski et al. 2007).

*Septic, Borrelia-induced, and reactive arthritis.* In several types of arthritis, MMP inhibition may reduce joint destruction.

Septic arthritis often leads to rapid destruction of joint cartilage and periarticular bone. Synovial fibroblasts exposed to *Staphylococcus aureus* show strong upregulation of several MMPs (Kanangat et al. 2006). MMP-7-deficient mice developed less joint destruction in a model of septic arthritis (Gjertsson et al. 2005). In another animal model, treatment with a bisphophonate together with antibiotics led to reduced bone resorption (Verdrengh et al. 2007). Although bisphosphonates inhibit resorption directly, it is possible that some of the effects may also have been mediated by the MMP-inhibitory action of the bisphophonate, because addition of corticosteroids to antibiotics and alendronate further improved some of the parameters under study. Furthermore, dexamethasone was shown to reduce duration of symptoms and long-term joint dysfunction in a randomized placebo-controlled study of 123 children with septic arthritis (Odio et al. 2003). Being strongly anti-inflammatory and known to inhibit several mediators, including, for example, MMP-9 (Liu et al. 2008), corticosteroids require further study in septic arthritis. Although not uncontroversial, if effective, they could revolutionize the treatment of septic arthritis.

Investigation of *Borrelia* arthritis has shown the presence of several MMPs and the aggrecanase ADAMTS-4 in the joint fluid of arthritis patients and in cartilage explants infected with *Borrelia* (Hu et al. 2001, Behera et al. 2006). These studies also showed that the MMP inhibitor batimastat reduces glucosaminoglycan and collagen degradation in cartilage explants infected with *Borrelia.* Based on its antimicrobial effect, doxycycline is already in widespread clinical use for *Borrelia* arthritis. In view of the MMP-inhibitory action of doxycycline, it would be interesting to compare the joint-sparing effect of doxycycline with that of other antibiotics in use for *Borrelia* arthritis. In reactive arthritis, however, randomized controlled trials have so far not shown any positive effects of tetracyclines (Lauhio et al. 1991, Smieja et al. 2001).

### Conclusion

MMPs are important mediators of connective tissue breakdown. They constitute promising pharmacological targets for clinical applications in orthopedics, rheumatology, and primary care. Furthermore, MMPs and related proteins may have a diagnostic role in prediction of the outcome of disease processes and healing.
